# Manipulating nanostructure to simultaneously improve the electrical conductivity and strength in microalloyed Al-Zr conductors

**DOI:** 10.1038/s41598-018-24527-4

**Published:** 2018-04-18

**Authors:** S. Y. Jiang, R. H. Wang

**Affiliations:** School of Materials Science and Engineering, Xi’an University of Technology, Xi’an, 710048 P.R. China

## Abstract

To elude the strength-electrical conductivity trade-off dilemma, a nanostructuring strategy was achieved in microalloyed Al-0.1wt.% Zr conductor by optimizing the processing route, leading to enhanced strength and simultaneously improved electrical conductivity. The nanostructural design involved ultrafine grains with coherent Al_3_Zr nanoprecipitates dispersed within the grain interior. The key is to create intragranular coherent Al_3_Zr nanoprecipitates with size of ~6 nm, which not only produce the highest precipitate hardening but also minimize the local strain field to reduce the scattering of electron motion. According to the targeted nanostructures, the processing route was revised to be artificially aged before cold drawing, instead of the post-aging as traditionally employed. The underlying mechanisms for improvement in strength and electrical conductivity were respectively discussed especially in terms of the coherent Al_3_Zr nanoprecipitates. It was quantitatively revealed from a strengthening model that the intragranular Al_3_Zr precipitate hardening was the predominant strengthening mechanism. Experimental results from three-dimensional atom probe (3DAP) demonstrating the Zr atom distribution in matrix as well as the geometrical phase analysis (GPA) results of local strain fields around the precipitates provided evidences to rationalize the promotion in electrical conductivity. The nanostructuring strategy in conjunction with the revised processing route offer a general pathway for manufacturing high-performance Al conductors in large-scale industrial applications.

## Introduction

The rapid industrial development calls for metals that possess low weight, high strength and also high electrical conductivity. The Al-based alloys, with low density and relative high conductivity, are certainly among the best candidates for the conductor materials^1–3^. Unfortunately, the pure Al has quite low strength, which limits its extensive applications. Doping minor alloying elements, such as Mg, Si, or Cu, has been widely employed to improve the strength of Al, through solid solution strengthening and precipitation strengthening^3–5^. Other effective approaches to strengthen the Al alloys include cold working and grain refinement. However, these approaches will unexceptionally cause a remarkable decrease in conductivity^6^, due to the scattering of electrons at defects, solutes, precipitates, or grain boundaries. The mutually exclusive strength-conductivity correlation becomes a bottleneck that restricts the development of new Al conductor materials with high strength and simultaneously high conductivity.

To evade the strength-conductivity inverse correlation in Al alloys requires a novel strategy on microstructural design. Valiev *et al*. proposed^7,8^ an approach of intelligent nanostructural design of Al alloys that enables to increase the strength and simultaneously raise the electrical conductivity. The new nanostructuring strategy was based on a combination of grain refinement down to ultrafine length scale with accelerated formation of nanoprecipitates within the grain interior. The high strength was mainly contributed by grain boundary strengthening, dislocation hardening, and precipitate hardening; and the enhanced electric conductivity was associated with a promoted intragranular precipitation and concomitantly a very low content of solute atoms within the Al matrix. This nanostructural design has been successfully applied to an Al-Mg-Si alloy^7^, where the grain refinement was realized by using severe plastic deformation (SPD) at room temperature while the nanosized second phase precipitates was created via dynamic aging during following SPD processing at elevated temperatures. The key in such a successful application is the dynamic aging during SPD processing, *i.e*., the grain refinement and nanoparticle precipitation happened concurrently. This processing has obvious advantages over traditional post-aging after SPD treatment: (a) the post-aging will greatly reduce the dislocations that are produced during SPD, leading to insufficient strength; and (b) the SPD-treated metals generally have ultrafine- or nano-grained structures with grain boundaries in high energy. During subsequent aging, precipitates are apt to nucleate at grain boundaries rather than within grain interior. The intergranular precipitation has been extensively observed during post-aging in ultrafine grained Al-Cu^9,10^, Al-Mg-Si^11^, and Al-Zn-Mg alloys^12,13^.

The aforementioned nanostructuring strategy is especially desirable for the Al alloys used as electrical conductors, where high strength and high electrical conductivity are simultaneously required. In industry, however, the Al electrical conductors are usually produced by following the classic processing: firstly subjected to solid solution, subsequently cold-drawn to wires, and finally exposed to artificial aging. The SPD processing (cold drawing) and aging treatment are separated, unlike the coupling (or dynamic aging during SPD) developed in Valiev *et al*.’s approach. How to achieve a similar nanostructuring design within the classic and economical Al alloy conductor processing remains a challenge, which, once realized, will definitely promote the further development of electrical conductors as well as the electrical transmission field.

In this paper, we perform systematic studies in microalloyed Al-Zr alloy conductors to reach a great improvement in the strength-conductivity combination by tailoring the microstructures. It is well known that Zr is one of the most effective alloying elements for Al alloys^14,15^. The Al-Zr system exhibits particular promise for developing thermally stable Al electrical conductors^16^. During aging treatment, Al_3_Zr precipitates will be nucleated from the Al-Zr solid solution, which, with a metastable cubic L12 structure, are highly stable at high homologous temperatures. The key is then to control the Al_3_Zr precipitation. Here, taking the present Al-Zr alloy conductors as an example, a revised processing route is proposed to fulfill the microstructure of submicron grains with nanosized precipitates embedded in the grain interior, which leads to enhanced strength and simultaneously improved conductivity. This revised processing route is expected to be equally effective for other heat-treatable Al alloy electrical conductors, such as Al-Mg-Si system.

## Results

### Microstructures of the Al-Zr conductors subjected to different processing routes

**Figure 1:**
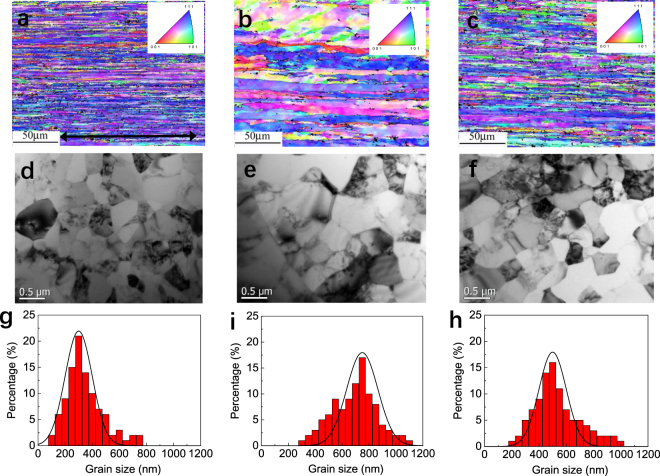
Representative EBSD images, TEM images, and statistical results on the grain size distribution. (**a**–**c**) Are longitudinal EBSD images, (**d**–**f**) are cross-sectional TEM images, and (**g**–**i**) are statistical results on the grain size distribution of the I-type (**a**,**d**,**g**), II-type-265 (**b**,**e**,**h**), and III-type-265 (**c**,**f**,**i**) Al-Zr conductors, respectively. The arrow in (a) indicates the cold drawing direction.

Figure 1a–c representatively show longitudinal EBSD images of the three kinds of Al-Zr conductors subjected to different processing routes, *i.e*., (i) with no heat treatment (I-type Al-Zr), (ii) with traditional processing route aged at 265 °C for 24 h (II-type-265 Al-Zr); and (iii) with revised processing route 265 °C for 24 h (III-type-265 Al-Zr), respectively. Details about the processing routes can be referred to the later section of Materials and Methods. All the three Al-Zr conductors have fibrous grains along the longitudinal direction. It is evident that the grains in the II-type-265 Al-Zr conductor are larger than in either the I-type or III-type-265 Al-Zr conductors. The larger grains in the former conductor are related to the traditional post-aging treatment after cold drawing imposed on the II-type Al-Zr conductor, where grains were thermally driven to coarsen. In comparison, no thermal annealing was applied to the I-type Al-Zr conductor. While in the III-type Al-Zr conductor, the artificial aging treatment was carried out before final cold drawing.

Figure 1d–f show the cross-sectional TEM images of the three Al-Zr conductors, representatively, to demonstrate the grain size. One can see that the grains in cross section are approximately equiaxed in all the three conductors. Again, the grains in the II-type-265 Al-Zr conductor are found to be larger than in the other two conductors from the TEM examinations. Quantitative statistics of the grain size, derived from the cross-sectional TEM examinations, are shown in Fig. 1h–j for comparison. The II-type-265 Al-Zr conductor has a broader grain size distribution with an average grain size of about 750 nm. The average grain size, by contrast, is about 300 nm and 500 nm in the I-type and III-type-265 Al-Zr conductors, respectively. Besides, the transverse grain size was also measured from longitudinal EBSD examinations, which was about 270 nm, 720 nm, and 460 nm respectively in the I-type, II-type, and III-type Al-Zr conductors. The transverse grain size derived from longitudinal EBSD measurements is roughly equal to (but slightly smaller than) that derived from cross-sectional TEM measurements. In a word, all the three kinds of Al-Zr conductors fall within the ultrafine grained regime, meeting the first requirement of the aforementioned nanostructuring strategy.

**Figure 2:**
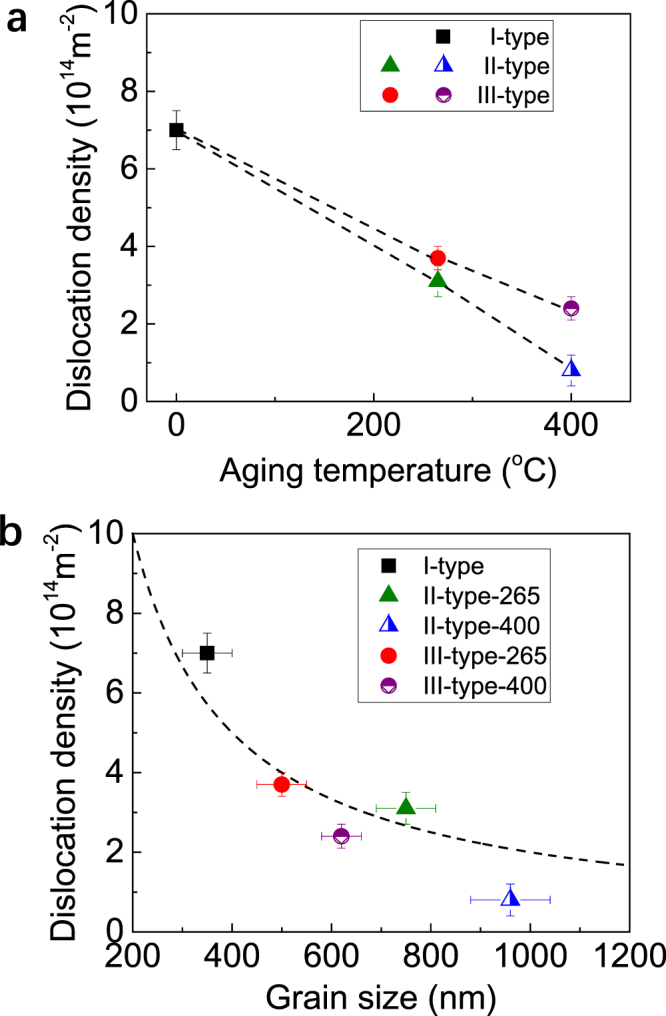
Experimental measurements on the dislocation density and dislocation density-grain size correlation. (**a**) Dislocation density of the Al-Zr conductors under different processing routes (I-type with no aging, II-type with post-aging after cold drawing, and III-type with pre-aging before cold drawing) and different aging temperature (265 and 400 °C). The aging temperature of 0 corresponds to I-type with no aging. (**b**) Correlation between the dislocation density ($\rho$) and the average grain size (*d*). The dash line follows the expression of *d* ∝ (*ρ*)^−1^.

The evolution in grain size related to the severe plastic deformation (cold drawing) and annealing treatment is definitely accompanied with an evolution in the dislocation density, since the grain refinement as well as grain coarsening are associated with the dislocation activities. The experimentally measured dislocation density is shown in Fig. 2a for the three kinds of Al-Zr conductors, *i.e*., the I-type one without artificial aging, the II-type ones with post-aging after cold drawing, and the III-type ones with pre-aging before cold drawing. Application of artificial aging treatment, either before or after cold drawing, always reduces the dislocations. The reduction in dislocations can be well understood since a great part of dislocations will be annihilated, as a result of accelerated dislocation movement and interactions under thermal activation. It is interesting that the dislocation density in the III-type-265 conductor (pre-aged at 265 °C) conductor is only slightly greater than in the II-type-265 one (post-aged at 265 °C), while the difference between the III-type and the II-type ones is remarkably broadened at the aging temperature of 400 °C. These imply that, in the present Al-Zr conductors aged at a relative low temperature such as 265 °C, the dislocation density is insensitive to the thermal history (pre-aging or post-aging) and mainly dominated by the accumulative plastic deformation (initially high temperature extrusion plus cold drawing). When the aging temperature is raised to a high one such as 400 °C, the thermal history plays a much more important role in determining the final dislocation density. The post-aging after cold drawing, rather than the pre-aging before cold drawing, will result in a highly reduced dislocation density. Figure 2b further demonstrates the correlation between the dislocation density and the grain size. Generally, the dislocation density varies inversely with the grain size. Starink *et al*. have proposed a model to predict grain refinement induced by cold severe plastic deformation^17^, which was successfully applied to about twenty Al alloys. A simple expression has been derived^17^ that the grain size *d* is inversely proportional to the dislocation density *ρ* to a first approximation, *i.e*., *d* ∝ (*ρ*)^−1^. Our experimental results are broadly reproduced by this expression, see the dash curve in Fig. 2b. Note that the II-type Al-Zr conductor post-aged at 400 °C (II-type-400) deviates far from the curve. The reason is mainly related to the high temperature annealing after cold drawing, which is much different from the prerequisite of cold severe plastic deformation in the above model.

**Figure 3:**
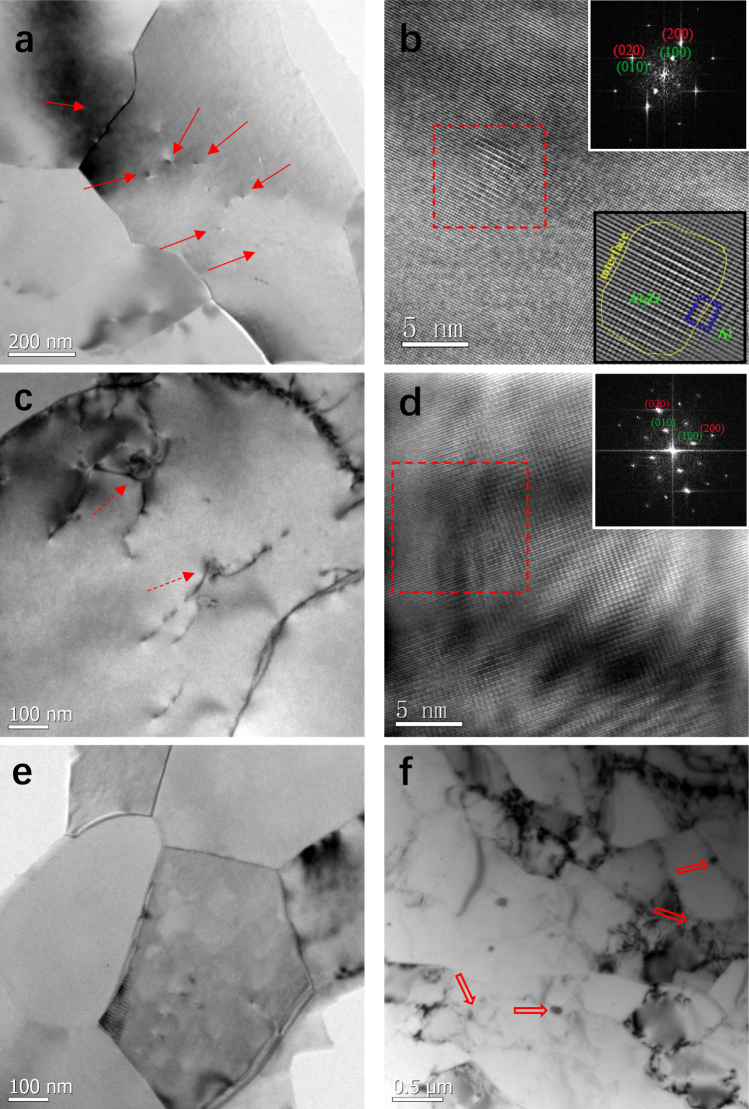
Representative TEM and HRTEM images of the Al-Zr conductors with different processing routes. (**a**) And (**b**) are TEM and HRTEM images to show the fine coherent Al_3_Zr precipitates (marked by solid arrows) within the grain interior in the III-type-265 Al-Zr conductor, respectively. (**c**) And (**d**) are TEM and HRTEM images to show the coarse and incoherent Al_3_Zr precipitates (marked by dash arrows) in the III-type-400 Al-Zr conductor, respectively. The FFT images respectively inserted in top-right corner of (**b**) And (**d**) are used to identify the Al_3_Zr precipitate with L1_2_ structure, and the IFFT image inserted in the bottom-right corner of (**c**) to demonstrate the coherent interface between the Al_3_Zr precipitate and Al matrix, where a typical closed Burgers circuit at the interface shows no misfit dislocations. Note the FFT and IFFT images are corresponding to the region marked by a red dash box. (**e**) is a TEM image of II-type-265 Al-Zr conductor where no precipitates are observed. (**f**) Is a TEM image to show intergranular Al_3_Zr precipitates (marked by open arrows) in the II-type-400 Al-Zr conductor.

The greatest difference in microstructure among the three kinds of Al-Zr conductors is the aging precipitation at nanosized length scale. In the I-type Al-Zr conductor, no nanosized precipitates were detected due to the absence of artificial aging treatment. In the III-type Al-Zr conductors, the precipitates were highly dependent on the aging temperature. A great number of ultrafine precipitates (with a number density of ~8 × 10^21^ m^−3^) were clearly found within the grain interior of the III-type-265 conductor that was pre-aged at 265 °C and subsequently cold-drawn (see Fig. 3a marked by arrows). Careful examinations manifested that these precipitates had a diameter close to ~6 nm, as representatively shown in a HRTEM image in Fig. 3b. The Fast Fourier Transformation (FFT) spectra corresponding to the region marked by a red dash box (as inserted in the top-right corner) help to identify the precipitate as Al_3_Zr with L1_2_ structure, which is similar to the characterization in a recent report^18^. Moreover, the corresponding inverse Fast Fourier Transformation (IFFT) with a typical closed Burgers circuit at the interface (inserted in the bottom-right corner) verify coherent interface between the Al_3_Zr precipitate and the matrix without misfit dislocations. While in the III-type-400 conductor that was pre-aged at 400 °C, the intragranular precipitates were still Al_3_Zr but in much sparser distribution (with a number density of ~2 × 10^13^ m^−3^) and much greater size (diameter > ~16 nm), see Fig. 3c,d. Most importantly, the Al_3_Zr precipitates in such a large size nearly lost coherence with the Al matrix (Fig. 3d). In the II-type Al-Zr conductors where post-aging was performed, complicated precipitation behaviors were observed, similarly depending on the aging temperature. Previous publications showed^9,10,12^ that the ultrafine grained Al alloys, exposed to artificial aging, were apt to nucleate precipitates at grain boundaries rather than in the grain interior. The intergranular precipitation is mainly attributed to the high energy characteristic of high-angle grain boundaries that are typical of ultrafine grained metals^9^. In order to minimize the grain boundary energy, solute atoms are ready to diffuse to and segregate at the grain boundaries. The solute concentration at grain boundaries is then considerably increased, finally triggering the nucleation of second phase particles^10^. Here in present II-type-400 conductor, some coarse Al_3_Zr precipitates were likewise found to locate at grain boundaries, and intragranular Al_3_Zr precipitates were hardly observed (Fig. 3f). In the II-type-265 conductor, by contrast, neither intergranular nor intragranular Al_3_Zr precipitates were detected at all (Fig. 3e). This phenomenon has been seldom reported. The possible reasons responsible for the absence of any precipitation include: (i) the solid solubility of solute elements will be greatly enhanced in a high-energy matrix such as processed by severe plastic deformation or presented in nanocrystalline^19^. The driving force for Zr atoms to precipitate and form intragranular Al_3_Zr particles is then insufficient in the cases of deficient Zr supersaturation as well as relatively low thermal activation of 265 °C aging temperature. (ii) The dislocation movements and interactions disturb the formation of Zr solute clusters and concomitantly the intragranular Al_3_Zr precipitates, provided the 265 °C-aging is not enough to stabilize the Zr clusters. (iii) The Zr diffusion coefficient in Al matrix is quite low^20^. Aged at the low temperature of 265 °C, the Zr diffusivity is too slow to segregate at the grain boundaries, restricting the intergranular precipitation. The above microstructural analyses on precipitation indicate that the dispersion of nanoparticles within grain interior can be only realize in the modified processing route with relative low aging temperature, *i.e*., in the III-type-265 Al-Zr conductor.

### Strength and electrical conductivity tailored by microstructures

**Figure 4:**
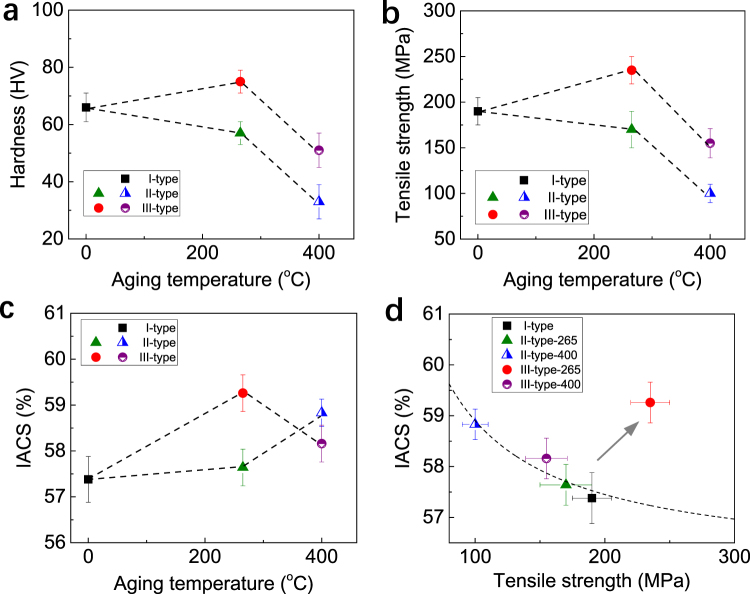
Hardness, tensile strength, and electrical conductivity of the Al-Zr conductors with different processing routes. Experimentally measured hardness (**a**), tensile strength (**b**), and electrical conductivity (**c**) of the Al-Zr conductors under different processing routes and different aging temperature (265 and 400 °C). (**d**) Electrical conductivity vs tensile strength of present Al-Zr conductors. The III-type-265 Al-Zr one clearly stands out, above and beyond the trend (represented by the dash curve) for the normal trade-off between strength and conductivity.

Accompanying with the evolution in microstructure induced by the different processing routes, there are significant changes in both the mechanical properties and electrical properties. Figure 4a–c show hardness, tensile strength, and electrical conductivity of the three kinds of Al-Zr conductors, respectively. The variation trend of Vickers hardness (HV) is completely consistent with that of tensile strength (σ), and a scaling relationship of σ ~ 3.0 × HV is roughly corroborated. This implies an equality between the two parameters in characterizing the mechanical property of present Al-Zr conductors. Compared with the I-type Al-Zr conductor free of artificial aging, only the Al-Zr conductor pre-aged at 265 °C (III-type-265) exhibited an enhanced hardness/strength, while all the other conductors unexceptionally displayed a reduction in the hardness/strength. The commonly observed reduction in hardness/strength is mostly related to a drop in the dislocation density and the grain coarsening motivated by thermal annealing. While in the III-type-265 conductor, the increase in hardness/strength is mainly attributed to the Al_3_Zr precipitate strengthening that is dominant over the negative effects of the reduced dislocations and coarsened grains. Lefebvre *et al*. reported experimental evidence that the transition point from precipitate shearing to bypassing mechanism was about 5–6 nm in diameter for the intragranular Al_3_Zr nano-precipitates^21^. In the present III-type-265 Al-Zr conductor, the dispersed Al_3_Zr nano-precipitates are almost uniform in size with a diameter of about 5–6 nm, indicative of the most strengthening response. In either the III-type-400 conductor with coarse Al_3_Zr precipitates in sparse distribution or the II-type-400 conductor with intergranular Al_3_Zr precipitates, the precipitate strengthening is not enough to make up the drop in strength related to the evolution in dislocation density and grain size.

**Figure 5:**
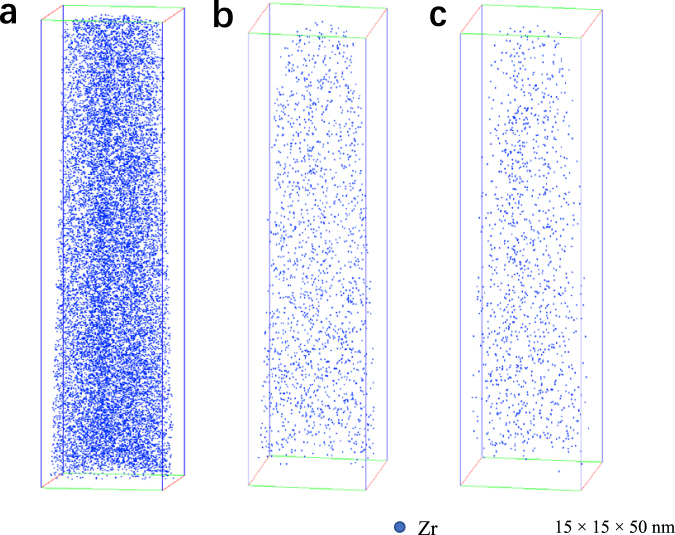
Representative 3DAP images to show the Zr atom distribution in the Al matrix in the I-type (**a**), II-type-400 (**b**), and III-type-265 (**c**) Al-Zr conductors, respectively. Only Zr atoms are shown in the 3DAP images for clearly revealing the Zr concentration.

**Figure 6:**
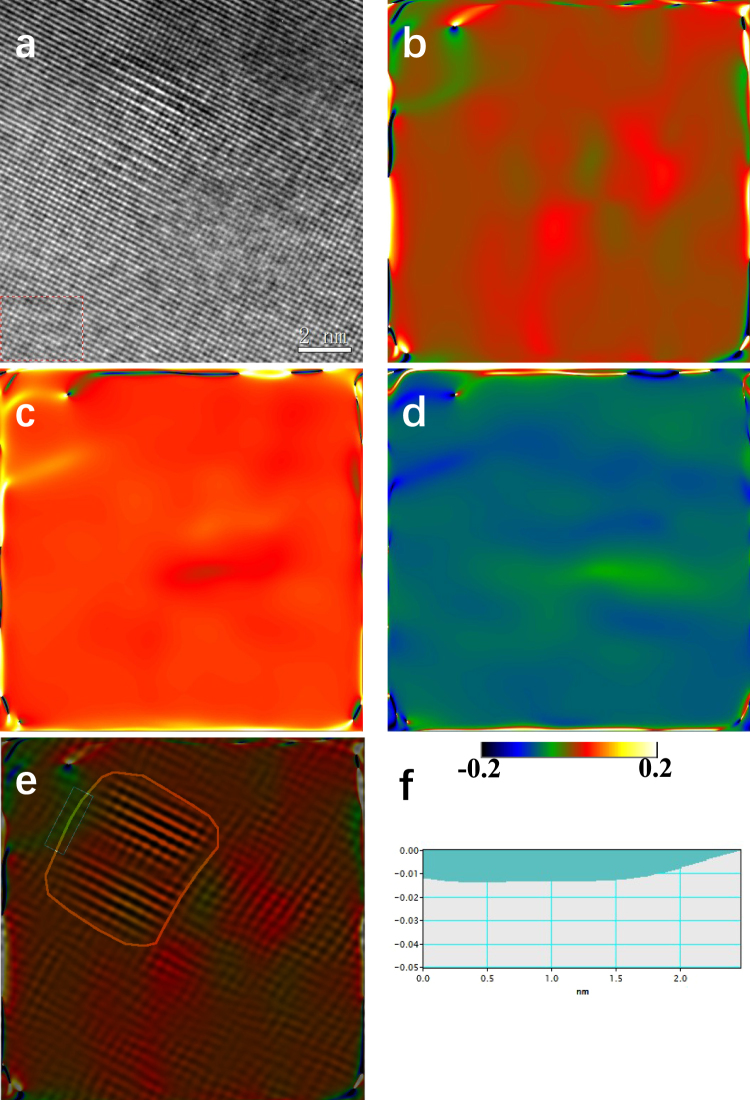
Geometrical phase analysis (GPA) of a coherent Al_3_Zr precipitate in the III-type-265 Al-Zr conductor. (**a**) A representative HRTEM image to show a coherent Al_3_Zr precipitate. Corresponding GPA results demonstrating the local ${\varepsilon }_{xx}\,$strain field (**b**), ${\varepsilon }_{yy}$ strain field (**c**), and ${\varepsilon }_{xy}$ strain field (**d**). (**e**) Is a picture overlapping the GPA ${\varepsilon }_{xx}\,$strain field with the HRTEM image, where the Al_3_Zr precipitate/matrix interface is highlighted by a loop line. (**f**) Shows the distribution of ${\varepsilon }_{xx}$ in a profile that was measured along a segment (as marked by green box in (**e**)) of the Al_3_Zr precipitate/matrix interface.

**Figure 7:**
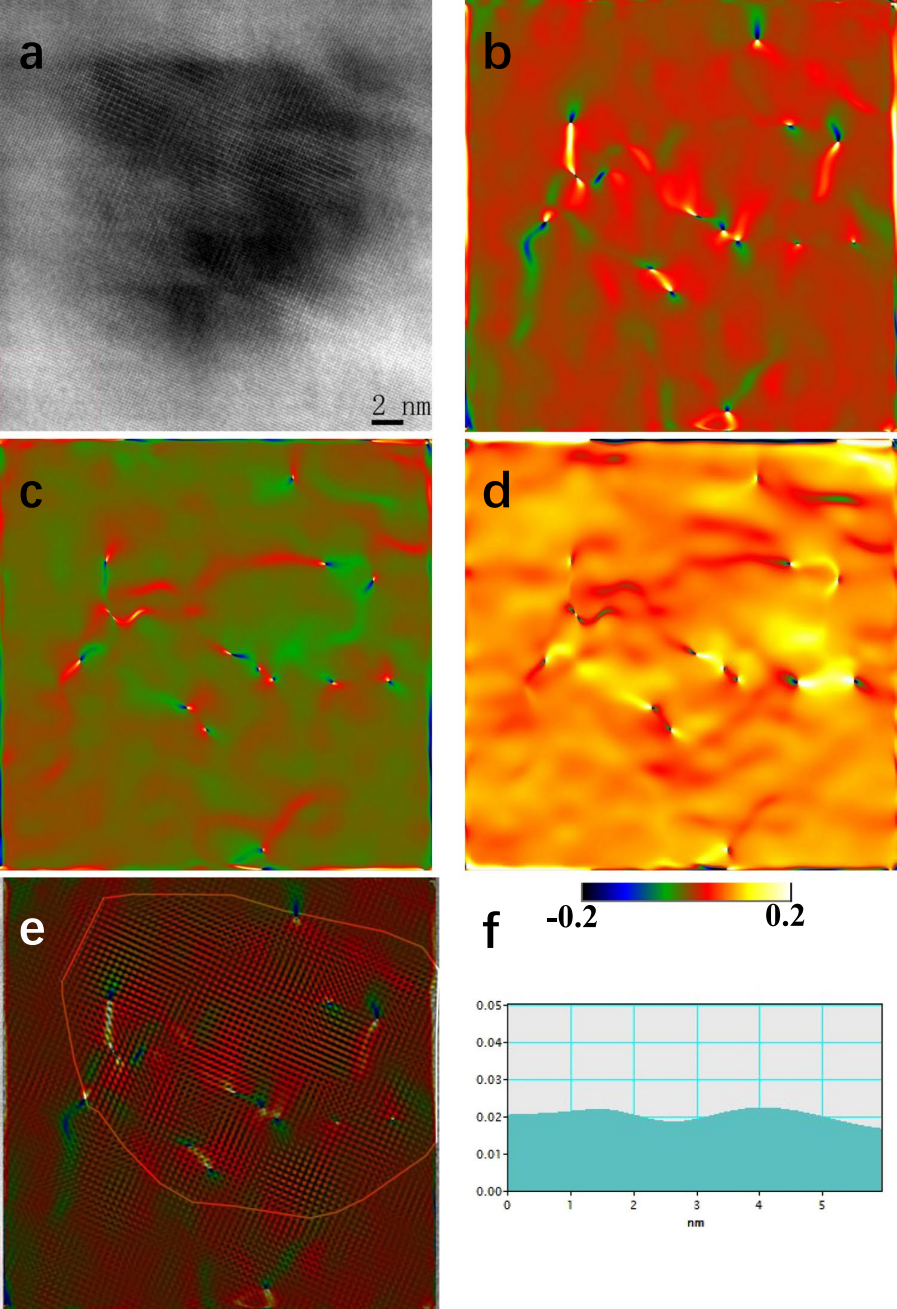
GPA of a semi-coherent Al_3_Zr precipitate in the III-type-400 Al-Zr conductor. (**a**) A representative HRTEM image to show a semi-coherent Al_3_Zr precipitate. Corresponding GPA results demonstrating the local ${\varepsilon }_{xx}\,$strain field (**b**), ${\varepsilon }_{yy}$ strain field (**c**), and ${\varepsilon }_{xy}$ strain field (**d**). (**e**) Is a picture overlapping the GPA ${\varepsilon }_{xx}\,$strain field with the HRTEM image, where the Al_3_Zr precipitate/matrix interface is highlighted by a loop line. (**f**) Shows the distribution of ${\varepsilon }_{xx}$ in a profile that was measured along a segment (as marked by green box in (**e**)) of the Al_3_Zr precipitate/matrix interface.

Generally, the reduction in strength is accompanied with an increase in electrical conductivity, and *vice versa*^22^. This is the well-known mutually exclusive strength-conductivity correlation. By comparing Fig. 4b,c, we can see that, in present work, all the other Al-Zr conductors followed this correlation except the III-type-265 one. The III-type-265 Al-Zr conductor exhibited not only the greatest hardness/strength, but also the highest electric conductivity. This makes the III-type-265 conductor clearly stand out, above and beyond the trend (represented by the dash curve in Fig. 4d) for the normal trade-off between strength and conductivity, see Fig. 4d. The nanostructuring strategy (ultrafine grain grains and nanosized precipitates dispersed within the grain interior) achieved in present Al-Zr conductors by pre-aging at low temperature instead of post-aging after cold drawing indeed resulted in enhanced strength and simultaneously improved electrical conductivity. Of special interest to note is that another key point impacting on the excellent strength-conductivity combination is the coherent characteristic of the Al_3_Zr precipitates elaborately created in the low-temperature pre-aging. The advantages of coherent Al_3_Zr precipitates in promoting the electrical conductivity mainly include: (i) the coherency of Al_3_Zr precipitate/matrix interfaces diminishes the energy barrier for precipitate nucleation^23^ and hence increases the number density of intragranular Al_3_Zr precipitates. Since a great number of Zr atoms are used for precipitate formation, the Zr atoms retained in the Al matrix are highly decreased. Figure 5a–c show representative three-dimensional atom probe (3DAP) images to demonstrate the Zr atom distribution within matrix in the I-type, II-type-400, and III-type-265 Al-Zr conductors, respectively. All the three images were captured under the same machine setting and in the same sizes of 15 × 15 × 50 nm. It was quantitatively measured that the Zr concentration in the matrix was about 0.027 ± 0.002 at.%, 0.007 ± 0.001 at.%, and 0.004 ± 0.001 at.% in the I-type, II-type-400, and III-type-265 Al-Zr conductors, respectively. These experimental results confirm the lowering in Zr concentration as a result of the Al_3_Zr precipitation. It is well known that the dissolved solute atoms decrease conductivity more than the nanosized precipitates^24^. The more are the Zr atoms consumed for precipitation, the higher is the electrical conductivity. (ii) The coherent Al_3_Zr precipitates produce only slight elastic strain in the around matrix, and the effect of local strain fields on electron scattering is hence relatively weak. The local strains can be evaluated by using geometrical phase analysis (GPA), which is a technique based on processing HRTEM micrographs^25–27^. The variation in the local lattice constant in the HRTEM micrographs is calculated via GPA by taking a strain free area as a reference. Despite its limitation of a relatively small field of view, the method has been used successfully to quantify strain fields around dislocations^28^, nanowires^29^, and more recently on precipitate-Al matrix interfaces^30^. Here in present work, GPA was similarly applied to compare the local strain induced by fully coherent Al_3_Zr precipitates and semi-coherent Al_3_Zr precipitates. Figure 6a shows a representative HRTEM image of a coherent Al_3_Zr precipitate in the III-type-265 Al-Zr conductor, with the results of GPA demonstrating the ${\varepsilon }_{xx}\,$strain field (Fig. 6b), ${\varepsilon }_{yy}$ strain field (Fig. 6c), and ${\varepsilon }_{xy}$ strain field (Fig. 6d). Figure 6e is a picture overlapping the GPA ${\varepsilon }_{xx}\,$strain field with the HRTEM image, where the Al_3_Zr precipitate/matrix interface is highlighted by a loop line. The distribution of ${\varepsilon }_{xx}$ at the Al_3_Zr precipitate/matrix interface (a segment as marked by green box in Fig. 6e) is measured in a profile, as shown in Fig. 6f. It is quantitatively revealed that the ${\varepsilon }_{xx}$ at interface is about – 1% for the coherent Al_3_Zr precipitate. In comparison, a semi-coherent Al_3_Zr precipitate taken from the III-type-400 Al-Zr conductor is analyzed in Fig. 7, including a representative HRTEM (Fig. 7a), corresponding GPA results (Fig. 7b–e), and ${\varepsilon }_{xx}$ profile at a segment of Al_3_Zr precipitate/matrix interface (Fig. 7f). The semi-coherent Al_3_Zr precipitate causes interfacial ${\varepsilon }_{xx}$ of about 2%, which is double of its coherent counterpart in absolute value. These GPA results and the comparison among them evidently manifest that the coherent Al_3_Zr precipitates dispersed in the III-type-265 Al-Zr conductor should have much less effect on the electron movement, leading to the highest electrical conductivity. While in the II-type-400 and III-type-400 Al-Zr conductors, the Al_3_Zr precipitates are all in large sizes, semi-coherent or incoherent, which produce stronger local strain fields and finally result in lower electrical conductivity by compared with the III-type-265 one.

## Discussions

The hardness/strength of present ultrafine grained Al-Zr conductors is contributed mainly by the solute atoms, forest dislocations, grain boundaries, and precipitates (only in the III-type-265 one). Correspondingly, the hardness (*H*) can be expressed as^31^

1&x2010;1 $$H={H}_{0}+{H}_{ss}+{H}_{d}+{H}_{gb},\,({\rm{for}}\,{\rm{other}}\,\mathrm{Al} \mbox{-} \mathrm{Zr}\,{\rm{conductors}})$$

1&x2010;2 $$H={H}_{0}+{H}_{ss}+{H}_{d}+{H}_{gb}+{H}_{p},\,({\rm{for}}\,{\rm{the}}\,\mathrm{III} \mbox{-} \mathrm{type} \mbox{-} \mathrm{265}\,{\rm{one}})$$

Where *H*_0_ is the base hardness of the pure Al that is arisen from friction stress (taken as 10 HV^32^), *H*_*ss*_, *H*_*d*_, and *H*_*p*_ are the contributions by solid solution hardening, forest dislocation hardening, grain boundary strengthening, and precipitate hardening, respectively.

The strength related to solid solution hardening (*σ*_*ss*_) is evaluated by^33^:

2 $${\sigma }_{ss}={k}_{{Zr}}{C}_{Zr}^{2/3},$$

Where ${C}_{Zr}$ is the Zr concentration dissolved in the matrix (in wt.%) and ${k}_{Zr}$ is a scaling factor (~63.0 MPa (wt.%)^−2/3^)^34^. The strength associated with the forest dislocation hardening (${\sigma }_{d}$) is given by^35^:

3 $${\sigma }_{d}=M\alpha Gb\sqrt{\rho },$$

where $M$ is the Taylor factor (∼3.0)^36^, $\alpha \,$is a constant with a value of approximately 0.14, $\rho$ is the dislocation density that is experimentally measured as shown in Fig. 2a, *G* and *b* are the shear modulus (28 GPa)^6^ and Burgers vector (0.286 nm)^6^ of Al, respectively. The strength induced by grain boundary hardening (${\sigma }_{gb}$) follows the well-known Hall-Petch relationship:

4 $${\sigma }_{gb}={k}_{HP}{d}^{-1/2},$$

with ${k}_{HP}$ a scaling constant that is taken as 0.042 MPa/m^−1/2^ for calculation. In the III-type-265 conductor, the precipitate hardening is expressed by^21^

5 $${\sigma }_{P}=\frac{0.4\,MGb}{\pi \lambda }\,\frac{\mathrm{ln}(\pi \,r/2b)}{\sqrt{1-\nu }},$$

where *r* and *λ* are mean radius and inter-particle spacing of the Al_3_Zr precipitates, v is the Poisson coefficient (~0.345), and other parameters have been defined before.

**Figure 8:**
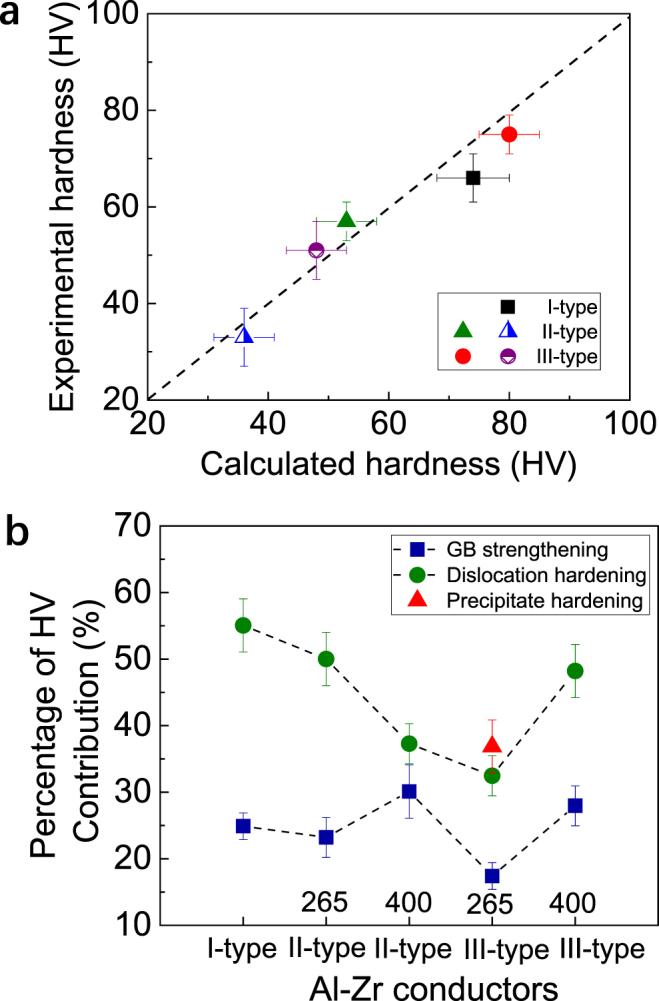
Calculations on the strengthening contributions. (**a**) Calculation on the total hardness of the Al-Zr conductors, in comparison with the experimental results. (**b**) The respective contribution of dislocation hardening, grain boundary strengthening, and precipitate hardening to the total hardness (in percentage, %) based on the calculations.

It is generally considered that the strength is about three times of the hardness HV^37^. This means that ${H}_{i}\approx {\sigma }_{i}/3$, with *i* = *ss*, *d*, *gb*, or *p*. The hardness of the Al-Zr conductors can then be respectively calculated by using equation (1) together with equations (2–5), based on the experimental measurements on the grain size, dislocation density, precipitate radius, *etc*. The calculations on hardness are plotted in Fig. 8a to compare with the experimental results. It is evident that the calculations are in broad agreement with the experimental data. Figure 8b further shows respective contribution of the different strengthening mechanisms to the hardness. One can see that dislocation hardening is basically the predominant strengthening mechanism in the Al-Zr conductors except in the III-type-265 one, where the precipitate hardening is slightly greater than the dislocation hardening. Besides, the grain boundary strengthening also plays an important role.

According to Mattiessen’s rule, the total resistivity of the Al-Zr conductors (${\rho }_{total}$) can be expressed by^22^

6 $${\rho }_{total}={\rho }_{t}+{\rho }_{i}+{\rho }_{d}$$

Where *ρ*_*t*_, *ρ*_*i*_, and *ρ*_*d*_ are the contributions from thermal vibrations, impurities, and lattice defects, respectively. The solute Zr atoms in the Al matrix act as impurity centers for the scattering of electron motions and hence significantly degrade the electrical conductivity. This is the main reason that I-type and II-type-263 Al-Zr conductors display the low electrical conductivity, because no Zr precipitation is existed in the two materials. The II-type-400 and III-type-400 conductors, although showing some degree of Al_3_Zr precipitation, both have a great value in ${\rho }_{d}$, because the Al_3_Zr precipitates are in a relatively low number density (most Zr atoms still dissolved in matrix) and large size (producing large local strain field as representative shown in Fig. 7). By contrast, the III-type-265 Al-Zr conductor underwent full Zr precipitation to create coherent Al_3_Zr precipitates, leading to low ${\rho }_{d}$ as well as low ${\rho }_{i}$. As a result, the III-type-265 Al-Zr conductor possesses the highest electrical conductivity in the case of greatest strength within the studied range here. This is substantially benefitted from the nanostructuring strategy that is achieved by a modified processing route of pre-aging before cold drawing, instead of the post-aging as generally employed in industrial applications.

## Methods

Microalloyed Al-0.1 wt.% Zr (abbreviated Al-Zr) conductors were used for present study. The Al-Zr alloys were melted and cast in a steam argon, by using 99.7 wt% industry pure Al and Al-10 wt.% Zr master alloy. The cast ingots were cut to the sizes of 20 × 20 × 200 mm and subsequently extruded to coiled materials with a diameter of 9.5 mm by using the LJ300 extrusion forming machine. The wheel revolving speed was 30 rpm and the extrusion temperature was ~500 °C. After this step, three different treatment routes were respectively applied for comparison. The first one included no any other heat treatments but merely cold-drawn to conductors (wires) in a diameter of 3.84 mm at room temperature. We define these Al-Zr conductors as I-type Al-Zr. The second route was the traditional one that followed solid solution at 550 °C for 48 h (with cold water quenching), subsequently cold-drawing to conductors (wires) similarly with 3.84 mm in diameter, and finally aging at 265 °C for 24 h (called II-type-265 Al-Zr) or at 400 °C for 24 h (II-type-400 Al-Zr). The third route was a revised one, where artificial aging was performed before cold drawing. The sequence was as follows: solid solution at 550 °C for 48 h (with cold water quenching), subsequently aging at 265 °C for 24 h (called III-type-265 Al-Zr) or at 400 °C for 24 h (III-type-400 Al-Zr), and finally cold-drawing to conductors (wires) again with a diameter of 3.84 mm. Note that the difference between the traditional route (II-type) and the revised route (III-type) lies in the order of artificial aging treatment, *i.e*., after or before the cold drawing to conductors, respectively. This difference, as discussed in the Results section, has remarkable effect on the microstructure and hence on the mechanical and electrical properties of the Al-Zr conductors.

Microstructures of the Al-Zr conductors were characterized by using electron backscattered diffraction (EBSD). The examinations were performed on a high-resolution JSM 7001F fitted with a Pegasus XM2–EBSD system that was operated at 20 kV. Specimens for EBSD examinations were prepared by electro-polishing. The electrolyte consisted of 25% nitric acid and 75% methanol at 253 K (–20 °C) for 1 min at an operation voltage of 15 V. The EBSD analyses were conducted on the planes parallel and perpendicular to the drawing direction, respectively.

Nanoscaled microstructures of the Al-Zr conductors were using transmission electron microscope (TEM) which was performed on a JEOL-2100 microscope operating at 200 kV. The TEM foils were prepared following the standard electro-polishing techniques for Al alloys^38,39^. Cross-sectional TEM examinations were carried out to measure the average grain size (*d*) of the conductor by using a linear intercept method, for which at least 150 grains were examined to obtain an average value. In measuring the size of precipitates, the smallest size ${l}_{1}$ and the largest size ${l}_{2}$ were first measured, and the precipitate size was determined as $l=\sqrt{{l}_{1}{l}_{2}}$^40,41^. At least 200 precipitates were examined to obtain the average precipitate size. The precipitate number density (${\varrho }$) was evaluated in term of the measure inter-particle spacing $\lambda$: ${\varrho }={\lambda }^{-3}$^40,41^. Similarly, at least 200 precipitates were measured to determine the average inter-particle spacing. More details about the measurements of precipitates were referred to some previous publications^38,39^. High resolution TEM (HRTEM) was used to image the atomic columns of the matrix in the close vicinity of the precipitates. Image analysis was performed by geometrical phase analysis (GPA), where the reference area used in the digital processing as a strain-free area was chosen in the matrix as far as possible from the precipitate under study, as a general procedure. On the images the standard deviation was never allowed to exceed 0.2%, which was then considered the maximum error on the measurements. More details can be referred to refs^25–28^.

Dislocation density was measured by performing X-ray diffraction (XRD) experiments. Each sample was tested at least six times to obtain a set of diffraction profiles. The evaluation of these profiles was done following the Multiple Whole Profile (MWP)-fit method developed by Ungar and co-workers^42,43^, where simulated profiles are fitted to the recorded profiles. This is done for all reflections simultaneously with ab initio theoretical functions for the strain- and size-induced profile broadening. The reader can refer to reference^44^ for experimental details.

To visibly reveal the microalloying mechanism at atomic level, three-dimensional atom probe (3DAP) experiments were performed using an Image Scientific Instruments 3000HR local electrode atom probe (LEAP). The 3DAP sample blanks with a square cross-sectional area of approximately 300 × 300 μm2 and length of 1 cm were prepared by a combination of slicing and mechanical grinding. A two-step electropolishing was used for making tips from these blanks^45^. A 10.0 vol% perchloric acid in methanol solution was used for coarse polishing, and the final polishing was performed using a solution of 2.0 vol% perchloric acid in butoxyethanol. APT data collection using the electrical pulsing mode was performed at a specimen temperature of 30 ± 0.3 K, with a voltage pulse fraction (pulse voltage/steady-state DC voltage) of 20%, a pulse repetition rate of 200 kHz and a background gauge pressure of <6.7 × 10^−8^ Pa (5 × 10^−10^ torr).

The Vickers hardness (HV) was tested on a LECO Hardness Tester (LV700AT) under a weight of 5 kg and with a dwelling time of 10 s. Data provided in the following sections are an average of at least 9 measurements. Uniaxial tensile tests of the conductors were performed in a Shimadzu AG-X testing machine. The tensile specimens has a gauge length of 150 mm and were tested at a constant strain rate of 5 × 10^−4^/s at room temperature. The tensile axis was parallel to the drawing direction. The electrical resistivity of each samples (gauge 1200 mm, standard measurement length 1000 mm) was measured by a double direct current electric bridge at room temperature, with the resistivity converted into %IACS (IACS: International Annealed Copper Standard). The following relation was used to express in IACS units: IACS = ω_Al_/ω_Cu_ 100%, where ω_Al_ is the conductivity of the studied Al alloy in MS/m and ω_Cu_ is the conductivity of copper (58.0 MS/m).
